# Association between the albumin-to-lymphocyte ratio and short-term mortality in critically Ill pediatric patients: a retrospective cohort study and machine learning analysis

**DOI:** 10.3389/fped.2026.1795057

**Published:** 2026-05-25

**Authors:** Kai Chen, Huiyan Tang, Yuanyuan Ye, Jie Chen, Jie Liu

**Affiliations:** 1 Department of Pediatrics, Jiaxing Hospital of Traditional Chinese Medicine, Jiaxing, Zhejiang, China; 2 Department of Biology, Jiaxing Construction Industry School, Jiaxing, Zhejiang, China; 3 Department of Critical Care Medicine, Jiaxing Hospital of Traditional Chinese Medicine, Jiaxing, Zhejiang, China

**Keywords:** albumin-to-lymphocyte ratio, machine learning, mortality, pediatric intensive care unit, risk stratification

## Abstract

**Background:**

Albumin-to-lymphocyte ratio (ALR), integrating nutritional status and immune-inflammatory response, has been proposed as a potential prognostic biomarker in various clinical settings. However, evidence regarding its prognostic value in critically ill pediatric patients remains limited. This study aimed to investigate the association between early ALR and short-term all-cause mortality in pediatric intensive care unit (PICU) patients and to evaluate its predictive utility.

**Methods:**

Clinical data were extracted from a large single-center PICU database including critically ill children admitted between 2010 and 2019. Patients were stratified into tertiles according to ALR measured within the first 24 hours after PICU admission. The primary outcome was 28-day all-cause mortality. Cox proportional hazards models were applied to assess the association between ALR and mortality after adjusting for potential confounders. Restricted cubic spline analyses were conducted to explore potential non-linear relationships. Kaplan–Meier survival curves were used to compare survival across ALR groups. Subgroup analyses were performed to evaluate the robustness of the association. In addition, multiple machine learning models incorporating ALR and other clinical variables were developed to predict 28-day mortality, and model performance was assessed using the area under the receiver operating characteristic curve (AUC).

**Results:**

A total of 7,591 critically ill children were included, with an overall 28-day mortality rate of 4.53%. Across ALR tertiles, the intermediate group showed the lowest observed 28-day mortality and the highest survival probability. In the fully adjusted Cox model, both the intermediate and highest tertiles were associated with lower mortality risk than the lowest tertile, with the strongest association observed in the intermediate tertile (T2: HR 0.55, 95% CI 0.42-0.74; T3: HR 0.65, 95% CI 0.49-0.86). Restricted cubic spline analysis revealed a significant non-linear association between ALR and mortality, characterized by a rapid decline in risk at low ALR levels, a nadir in the intermediate range, and a slight increase at higher ALR levels. This pattern was consistent with the Kaplan–Meier analysis, which showed the highest survival probability in the middle ALR tertile. Subgroup analyses showed consistent associations across different clinical strata. Among the machine learning models, the XGBoost model achieved the best discriminative performance, with an AUC of 0.835. In the SHAP-based interpretation, ALR contributed to model prediction and ranked tenth among the selected features.

**Conclusion:**

As a simple, readily available, and cost-effective biomarker derived from routine laboratory tests, ALR may serve as a complementary tool for early risk stratification and prognostic assessment in the PICU setting.

## 1 Introduction

The spectrum of pediatric diseases varies substantially across countries and regions. However, infectious diseases, malnutrition, and injuries remain major causes of childhood mortality. In recent years, global mortality among children under five years of age has declined overall, yet a large number of children still die each year from causes that are preventable or treatable (1, 2). In hospital settings, critically ill children are commonly admitted due to severe infections, acute respiratory failure, neurological emergencies, major trauma, and postoperative complications. Disease progression is often rapid, and organ dysfunction can develop and overlap within a short period of time. In some regions, the demand for intensive care is increasing, accompanied by growing medical complexity among pediatric patients (3) Therefore, early risk stratification and prognostic assessment at admission are essential for optimizing resource allocation in the pediatric intensive care unit (PICU) and for improving clinical outcomes.

Pediatric diseases are often characterized by complex immune and inflammatory responses accompanied by a rapid depletion of nutritional reserves. Under these conditions, single inflammatory markers or isolated nutritional risk scores are frequently insufficient to fully capture disease severity and the patient's physiological reserve. Recent studies have shown that several composite indices integrating inflammatory and nutritional components, such as the lactate to albumin ratio (4), prognostic nutritional index (5), red cell distribution width to albumin ratio (6), neutrophil percentage to albumin ratio (7), and hemoglobin, albumin, lymphocyte, and platelet score (8), may offer greater prognostic value than individual inflammatory markers alone.

Composite indicators that combine serum albumin and lymphocyte levels have been applied to prognostic assessment in a variety of diseases (9–11). However, clear evidence regarding the prognostic value of the albumin to lymphocyte ratio (ALR) in critically ill pediatric patients is still lacking. Given that critically ill children often experience pronounced immune and inflammatory activation together with nutritional derangements (12, 13), we hypothesized that ALR might also serve as a reliable prognostic biomarker in this population.

## 2 Methods

### 2.1 Data source

This study used clinical data from the PICU database. The database contains anonymized medical records of critically ill children admitted between 2010 and 2019. The first author, Kai Chen(Record ID 74764316), successfully completed the CITI program and was therefore granted access to the database.

### 2.2 Participants

**Figure 1:**
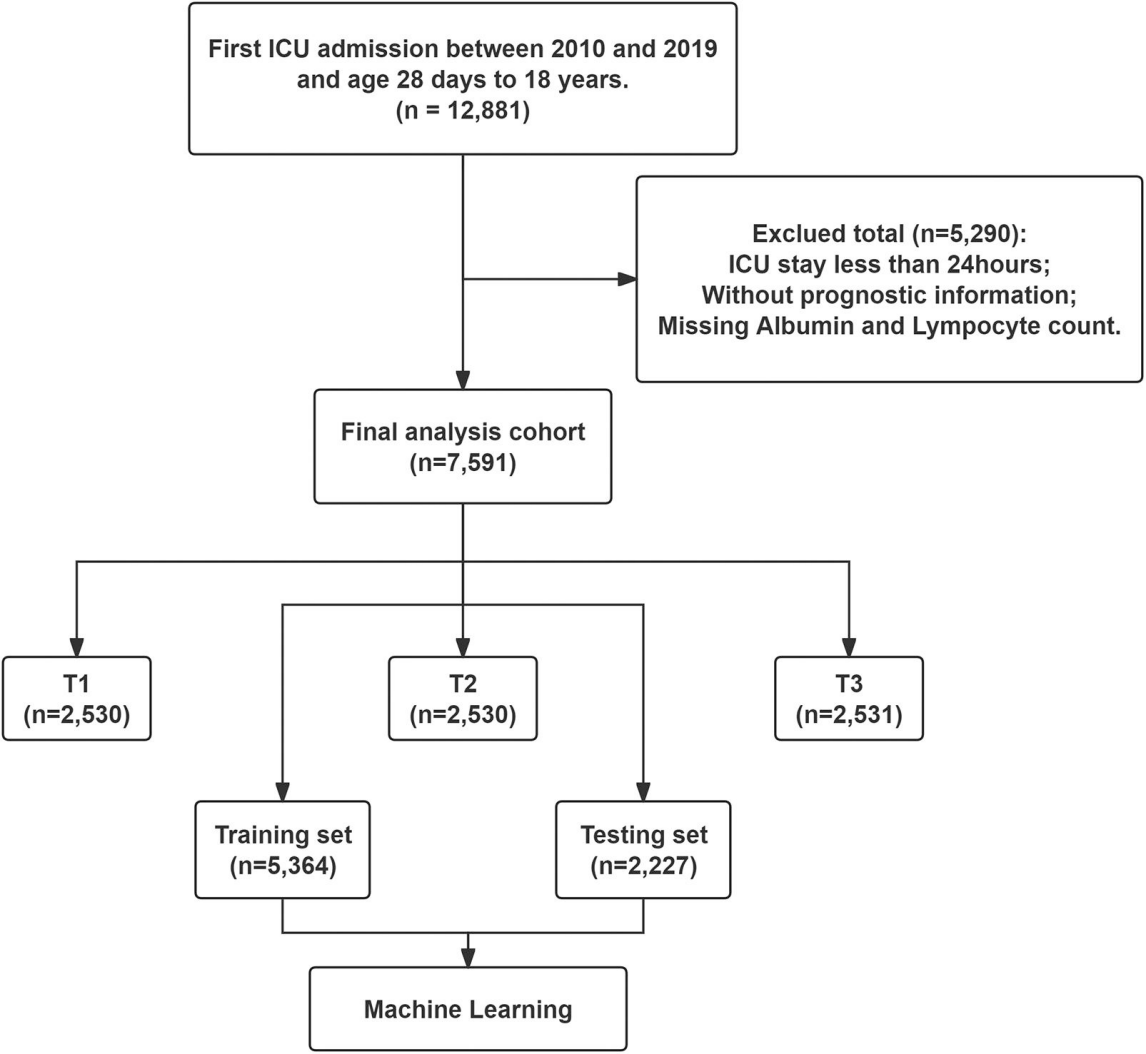
Study population selection and dataset partitioning for ALR-based mortality analysis in pediatric patients. Flowchart showing patient screening from the PIC database, application of prespecified exclusion criteria, formation of the final analysis cohort, tertile stratification according to ALR, and random partitioning of the dataset into training and testing sets for machine-learning analysis. Exclusions were applied jointly according to prespecified criteria; the excluded total was not broken down into mutually exclusive counts for each single reason. ALR, albumin-to-lymphocyte ratio; ICU, intensive care unit; PICU, pediatric intensive care unit; T1, first tertile; T2, second tertile; T3, third tertile.

Critically ill children admitted to the ICU for the first time were eligible for inclusion. Prespecified exclusion criteria included a PICU stay of less than 24 hours, missing key outcome information, and absence of essential laboratory data after admission, mainly including missing serum albumin or lymphocyte-related measurements. The <24-hour criterion was specified *a priori* because ALR and other baseline variables were defined using data obtained within the first 24 hours after PICU admission, and this criterion was intended to improve the comparability of baseline assessment. In the actual screening workflow, however, the excluded cohort reflected the joint application of prespecified exclusion criteria rather than mutually exclusive counts for each individual reason. The final study cohort was established accordingly. Patients were then stratified into tertiles based on early ALR levels at admission (Figure 1).

### 2.3 Data collection

Data extraction was performed using SQL with pgAdmin (version 4.1) to retrieve information from the PICU database. All variables were derived from the first recorded clinical data within the initial 24 hours after ICU admission. Specifically, data on demographic characteristics, vital signs, baseline comorbidities, and laboratory parameters were collected. A complete list of variables is provided in Supplementary Table S1. The ALR was calculated using the following formula: ALR = Albumin (g/L)/Lymphocyte count (10^9/L) (9). To address missing data and minimize potential bias, variables with more than 20 percent missing values were excluded. For the remaining variables with less than 20 percent missingness, multiple imputation by chained equations was conducted using the mice package in R.

### 2.4 Statistical analysis

Normality of continuous variables was examined with the Kolmogorov–Smirnov test. These data are reported as mean with standard deviation or as median with interquartile range, depending on distribution. Categorical variables are reported as numbers and percentages. Between-group differences were tested with the t test or one-way analysis of variance when variables followed a normal distribution. For skewed variables, the Mann–Whitney U test or the Kruskal–Wallis test was used. Kaplan–Meier(KM) curves were created to show mortality across ALR groups. Group differences were assessed with the log rank test.

To evaluate the link between ALR and all-cause mortality, we used Cox proportional hazards models to calculate hazard ratios with 95 percent confidence intervals. Potential confounders were included as needed. Covariates for the final multivariable models were chosen using a prespecified approach. Only variables selected by the Boruta method and justified by clinical relevance were retained. Model 1 included no covariates. Model 2 adjusted for age, sex, and race. Model 3 additionally adjusted for platelet count, potassium, sodium, pH, calcium, partial pressure of carbon dioxide (PCO2), triglycerides (TG), alanine, bilirubin, sepsis, neurological disease, and cardiovascular disease. Restricted cubic spline(RCS) regression was used to assess possible non-linear relationships between ALR and mortality.

To test the stability of ALR as a prognostic marker for all-cause mortality, we ran stratified analyses by baseline characteristics and then performed subgroup analyses. Statistical significance was set at a two-sided p value below 0.05. All analyses were conducted in R, version 4.4.2.

### 2.5 Construction and assessment of the prognostic models

In this study, we used the Boruta algorithm to evaluate the predictive value of ALR among candidate variables. Boruta is a robust feature selection method based on the random forest framework and is designed to identify all predictors that are significantly associated with the target outcome. The selected features were then incorporated into multiple machine learning algorithms to construct prognostic models. Initially, four algorithms were considered, including decision tree, k-nearest neighbors classifier (KNNC), random forest (RF), and extreme gradient boosting (XGBoost). The dataset was randomly divided into a training set (70%) and a testing set (30%).Within the training set, hyperparameter tuning was independently performed using a five-fold cross-validation strategy combined with grid search. The final models were subsequently trained on the entire training dataset using the optimized parameters and evaluated in the independent testing set. Model performance was primarily assessed by discrimination using the area under the receiver operating characteristic curve (AUROC), receiver operating characteristic (ROC) curves, and precision–recall (PR) curves. For the best-performing model, the ROC curve with 95% confidence interval (CI) was further generated, and the optimal cutoff value was identified from the ROC analysis, with the corresponding sensitivity and specificity reported. Calibration was additionally assessed using calibration plots and Brier scores. To enhance model interpretability, the SHAP method was applied to the optimal model.

## 3 Results

### 3.1 Baseline characteristics

A total of 7,591 critically ill children were included in this study and were categorized into three groups according to tertiles of ALR at admission. The median age of the cohort was 1.37 years, and 55.53 percent of the patients were male. The overall median ALR was 16.63, with an interquartile range of 11.04 to 25.83. ALR values increased sequentially across groups, with median ALR levels of 9.11 (6.85 to 11.04) in T1, 16.63 (14.66 to 19.02) in T2, and 31.70 (25.83 to 42.52) in T3. As ALR levels increased, the overall age of patients showed an upward trend, and the sex distribution also differed among the three groups. With respect to laboratory findings, patients in higher ALR groups generally had higher serum albumin levels, lower lymphocyte related indices, and a higher proportion of neutrophils, along with differences in selected liver and kidney function parameters and electrolyte profiles. The distribution of PICU types and primary diagnostic categories was not entirely consistent across ALR groups. Regarding outcomes, there were 344 deaths within 28 days, corresponding to an overall mortality rate of 4.53 percent. The 28 day mortality rates in T1, T2, and T3 were 6.17 percent, 3.00 percent, and 4.43 percent, respectively, with statistically significant differences observed among the groups (Table 1).

**Table 1:** Baseline characteristics and 28-day outcomes of pediatric patients stratified by ALR tertiles at pICU admission.

Characteristic	Overall (*n* = 7,591)	T1 (*n* = 2,530)	T2 (*n* = 2,530)	T3 (*n* = 2,531)	*p*-value
Age (year)	1.37 (0.39–4.37)	0.61 (0.21–1.63)	1.29 (0.45–3.71)	3.73 (1.20–8.12)	<0.001
Gender, n (%)					0.017
Female	3,376.00 (44.47%)	1,120.00 (44.27%)	1,178.00 (46.56%)	1,078.00 (42.59%)	
Male	4,215.00 (55.53%)	1,410.00 (55.73%)	1,352.00 (53.44%)	1,453.00 (57.41%)	
Race, n (%)					0.171
Han	7,520.00 (99.06%)	2,499.00 (98.77%)	2,511.00 (99.25%)	2,510.00 (99.17%)	
Others	71.00 (0.94%)	31.00 (1.23%)	19.00 (0.75%)	21.00 (0.83%)	
Hemoglobin (g/L)	107.00 (94.00–119.00)	105.00 (93.00–117.00)	106.00 (95.00–118.00)	108.00 (95.00–121.00)	<0.001
WBC (109/L)	9.19 (6.38–13.09)	11.33 (8.36–15.08)	8.71 (6.27–12.01)	7.48 (4.92–11.37)	<0.001
Lymphocyte count (109/L)	2.25 (1.47–3.31)	3.93 (3.28–5.19)	2.27 (1.97–2.58)	1.20 (0.89–1.49)	<0.001
Platelet (109/L)	266.00 (176.00–360.00)	318.00 (207.00–424.00)	269.00 (184.00–352.00)	227.00 (149.00–305.00)	<0.001
RBC (109/L)	3.88 (3.35–4.34)	3.79 (3.28–4.28)	3.91 (3.39–4.34)	3.91 (3.39–4.37)	<0.001
Neutrophils, n (%)	67.60 (52.10–78.70)	52.80 (38.50–65.60)	68.20 (56.50–76.70)	79.20 (69.20–86.30)	<0.001
Potassium	3.70 (3.30–4.00)	3.71 (3.30–4.20)	3.60 (3.30–4.00)	3.70 (3.30–4.00)	<0.001
Sodium (mmol/L)	138.00 (135.00–140.00)	137.00 (135.00–140.00)	138.00 (136.00–141.00)	138.00 (135.00–141.00)	<0.001
Calcium (mmol/L)	1.21 (1.12–1.27)	1.23 (1.14–1.29)	1.22 (1.13–1.28)	1.18 (1.10–1.24)	<0.001
Albumin (g/L)	37.70 (33.90–41.20)	35.70 (31.80–39.40)	38.20 (34.80–41.30)	38.90 (35.40–42.30)	<0.001
PCO2 (mmHg)	37.60 (32.50–43.40)	38.50 (32.60–45.50)	37.90 (32.90–43.20)	36.40 (32.30–41.60)	<0.001
PH	7.38 (7.32–7.43)	7.36 (7.30–7.42)	7.38 (7.33–7.43)	7.39 (7.34–7.44)	<0.001
PO2 (mmHg)	144.00 (77.60–185.00)	131.00 (72.10–177.00)	152.00 (83.80–186.00)	149.00 (78.80–188.00)	<0.001
TG (mmol/L)	0.81 (0.56–1.16)	0.96 (0.63–1.37)	0.76 (0.54–1.13)	0.75 (0.52–1.10)	<0.001
TC (mmol/L)	3.12 (2.41–3.78)	3.05 (2.37–3.72)	3.11 (2.39–3.74)	3.16 (2.48–3.85)	<0.001
Alanine (U/L)	20.00 (13.00–34.00)	22.00 (14.00–42.00)	19.00 (13.00–31.00)	20.00 (12.00–33.00)	<0.001
Cr (*μ*mol/L)	39.00 (33.00–48.00)	38.00 (32.00–46.00)	38.00 (32.00–46.00)	42.00 (34.00–52.50)	<0.001
Bilirubin (μmol/L)	10.20 (6.30–17.70)	10.20 (6.10–20.89)	10.30 (6.40–17.00)	9.90 (6.30–15.80)	<0.001
ALR	16.63 (11.04–25.83)	9.11 (6.85–11.04)	16.63 (14.66–19.02)	31.70 (25.83–42.52)	<0.001
ICU types, n (%)					<0.001
General ICU	1,277.00 (16.82%)	435.00 (17.19%)	348.00 (13.75%)	494.00 (19.52%)	
PICU	1,537.00 (20.25%)	556.00 (21.98%)	390.00 (15.42%)	591.00 (23.35%)	
CICU	2,218.00 (29.22%)	507.00 (20.04%)	942.00 (37.23%)	769.00 (30.38%)	
NICU	416.00 (5.48%)	289.00 (11.42%)	81.00 (3.20%)	46.00 (1.82%)	
SICU	2,143.00 (28.23%)	743.00 (29.37%)	769.00 (30.40%)	631.00 (24.93%)	
Sepsis, n (%)	176.00 (2.32%)	72.00 (2.85%)	39.00 (1.54%)	65.00 (2.57%)	0.005
Urinary, n (%)	102.00 (1.34%)	30.00 (1.19%)	37.00 (1.46%)	35.00 (1.38%)	0.679
Trauma, n (%)	543.00 (7.15%)	166.00 (6.56%)	180.00 (7.11%)	197.00 (7.78%)	0.240
Respiratory, n (%)	879.00 (11.58%)	367.00 (14.51%)	235.00 (9.29%)	277.00 (10.94%)	<0.001
Neurological, n (%)	646.00 (8.51%)	184.00 (7.27%)	187.00 (7.39%)	275.00 (10.87%)	<0.001
Neoplasm, n (%)	339.00 (4.47%)	92.00 (3.64%)	94.00 (3.72%)	153.00 (6.05%)	<0.001
Hematological, n (%)	242.00 (3.19%)	101.00 (3.99%)	59.00 (2.33%)	82.00 (3.24%)	0.003
Digestive, n (%)	680.00 (8.96%)	279.00 (11.03%)	229.00 (9.05%)	172.00 (6.80%)	<0.001
Congenital, n (%)	2,686.00 (35.38%)	802.00 (31.70%)	1,099.00 (43.44%)	785.00 (31.02%)	<0.001
Cardiovascular, n (%)	688.00 (9.06%)	211.00 (8.34%)	256.00 (10.12%)	221.00 (8.73%)	0.068
28-day mortality, n (%)	344.00 (4.53%)	156.00 (6.17%)	76.00 (3.00%)	112.00 (4.43%)	<0.001

ALR, albumin-to-lymphocyte ratio; T1, first tertile; T2, Second tertile; T3, Third tertile; WBC, White Blood Cell count; RBC, Red Blood Cell count; TG, Triglycerides; TC, Total Cholesterol; Cr, Creatinine; PCO2, Partial Pressure of Carbon Dioxide; PO2, Partial Pressure of Oxygen; PH, pH (Potential of Hydrogen); ICU, Intensive Care Unit; PICU, Pediatric Intensive Care Unit; CICU, Cardiac Intensive Care Unit; NICU, Neonatal Intensive Care Unit; SICU, Surgical Intensive Care Unit.

### 3.2 Clinical outcomes

**Figure 2:**
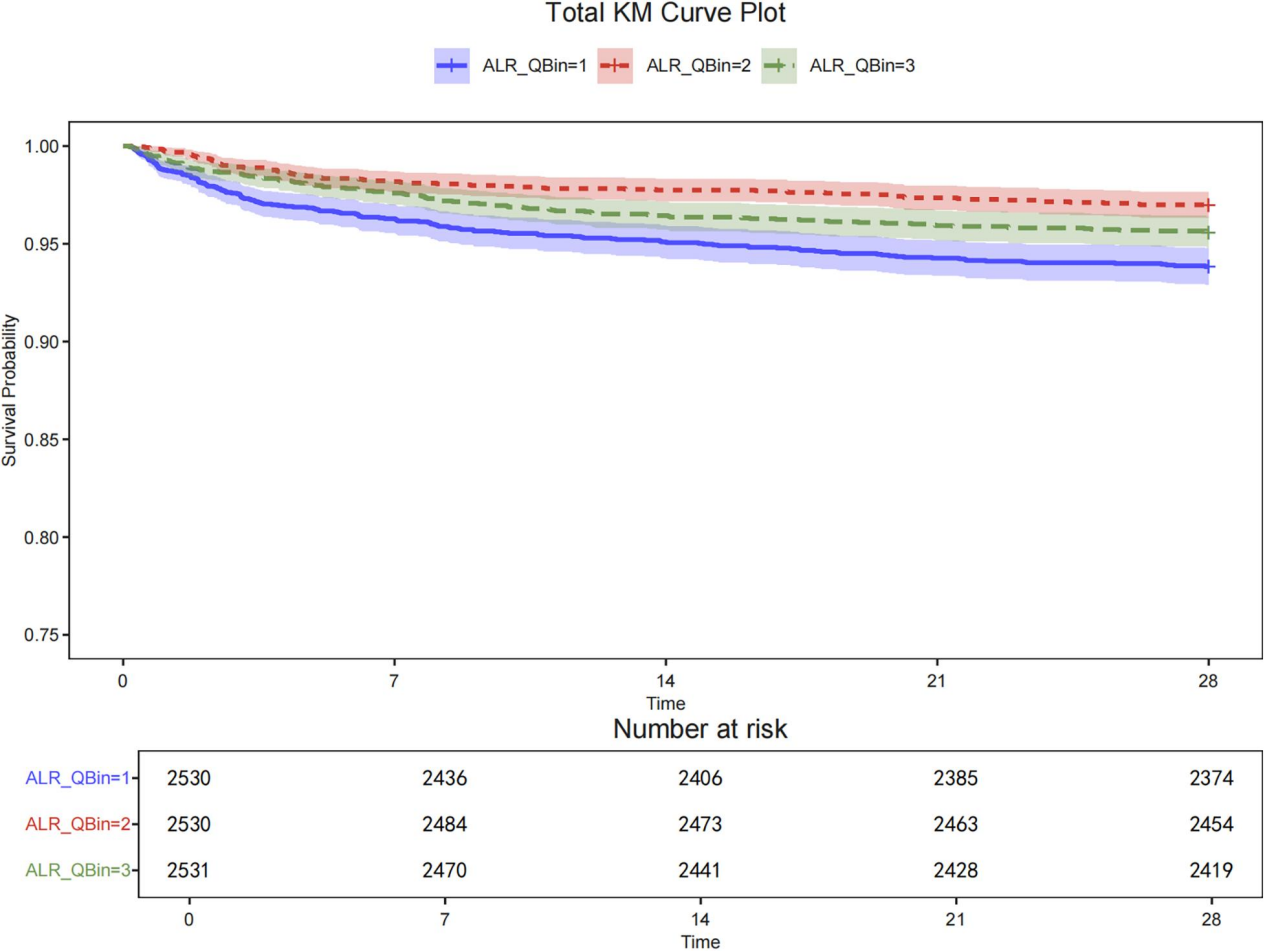
Association between ALR tertiles and 28-day mortality in pediatric patients using kaplan–meier survival analysis. Kaplan–Meier curves show 28-day survival probability across tertiles of ALR measured within the first 24 hours after PICU admission. Shaded areas indicate 95% confidence intervals. The numbers at risk are shown below the plot. Group differences were assessed using the log-rank test. A two-sided *P* < 0.05 was considered statistically significant. ALR, albumin-to-lymphocyte ratio; KM, Kaplan–Meier; T1, first tertile; T2, second tertile; T3, third tertile.

 Figure 2 presents the 28 day KM survival curves stratified by tertiles of ALR at admission. Overall, patients in the second tertile exhibited the highest survival probability, those in the first tertile had the lowest survival, and the third tertile showed intermediate survival. These findings indicate that 28 day survival outcomes differ across ALR levels.

A multivariable Cox proportional hazards model was applied to assess the relationship between ALR at admission and 28-day all-cause mortality. The results are presented in Table 2. When ALR tertiles were entered as a categorical variable, both T2 and T3 were associated with a lower risk of 28-day mortality compared with T1 across all models, with T2 showing the lowest HR. In Model 1, the HRs were 0.48 (95% CI 0.36 to 0.63, *p* < 0.001) for T2 and 0.71 (95% CI 0.56 to 0.91, *p* = 0.006) for T3. In Model 2, the HRs were 0.49 (95% CI 0.37 to 0.64, *p* < 0.001) for T2 and 0.71 (95% CI 0.55 to 0.93, *p* = 0.014) for T3. In Model 3, the HRs were 0.55 (95% CI 0.42 to 0.74, *p* < 0.001) for T2 and 0.65 (95% CI 0.49 to 0.86, *p* = 0.002) for T3. These findings indicate that the lowest mortality risk was observed in the intermediate tertile rather than in the highest tertile.

**Table 2:** Association between ALR tertiles and 28-day all-cause mortality in pediatric patients using Cox proportional hazards models.

Variables	Model 1		Model 2		Model 3	
	HR (95%CI)	*P*	HR (95%CI)	*P*	HR (95%CI)	*P*
ALR						
T1	1.00 (Reference)		1.00 (Reference)		1.00 (Reference)	
T2	0.48 (0.36∼0.63)	**<0**.**001**	0.49 (0.37∼0.64)	**<0**.**001**	0.55 (0.42∼0.74)	**<0**.**001**
T3	0.71 (0.56∼0.91)	**0**.**006**	0.71 (0.55∼0.93)	**0**.**014**	0.65 (0.49∼0.86)	**0**.**002**
HR for trend	0.82 (0.72∼0.93)	**0**.**003**	0.81 (0.71∼0.94)	**0**.**005**	0.79 (0.68∼0.91)	**0**.**001**

Model1: Crude.

Model2: Adjust: Age, Gender, Race.

Model3: Adjust: Age, Gender, Race, Platelet, Potassium, Sodium, PH, Calcium, PCO2, TG, Alanine, Bilirubin, Sepsis, Neurological, Cardiovascular.

HR, Hazard Ratio; CI, Confidence Interval; ALR, Albumin-to-Lymphocyte Ratio; T1, first tertile; T2, Second tertile; T3, Third tertile; WBC, White Blood Cell count; RBC, Red Blood Cell count; TG, Triglycerides; Cr, Creatinine; PCO2, Partial Pressure of Carbon Dioxide; PH, Potential of Hydrogen.

**Figure 3:**
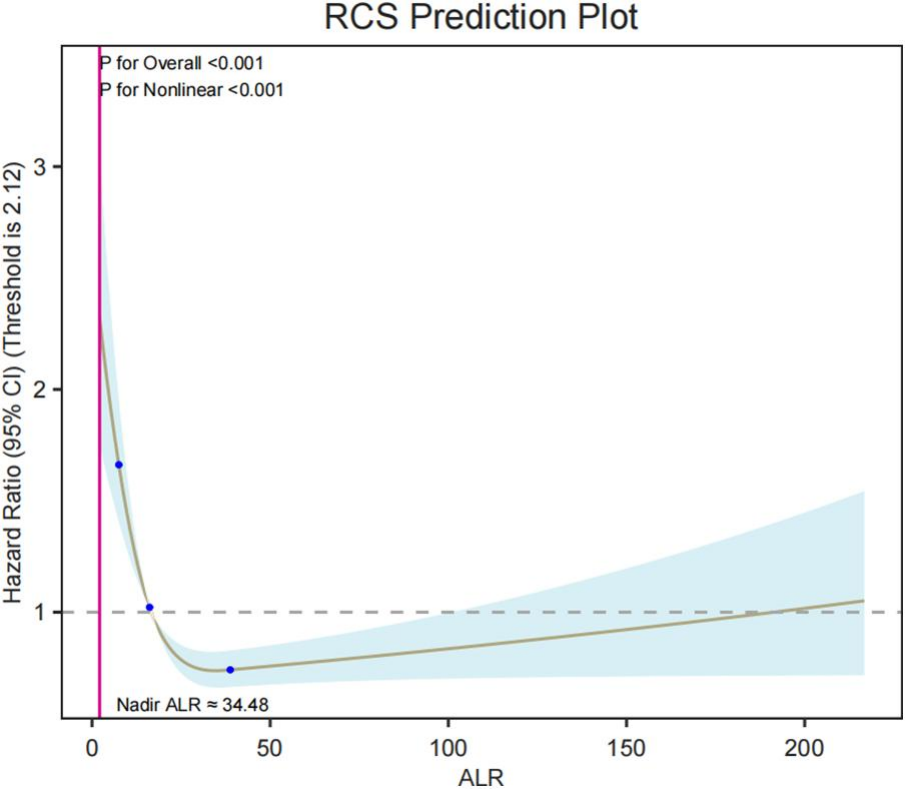
Association between ALR and 28-day mortality in pediatric patients using restricted cubic spline Cox regression. Restricted cubic spline (RCS) analysis showing the adjusted association between continuous ALR and 28-day all-cause mortality. The solid curve represents the adjusted hazard ratio (HR), and the shaded area represents the 95% confidence interval (CI). The horizontal dashed line indicates HR = 1.0. The model was adjusted for age, sex, race, platelet count, potassium, sodium, pH, calcium, partial pressure of carbon dioxide (PCO2), triglycerides (TG), alanine, bilirubin, sepsis, neurological disease, and cardiovascular disease, consistent with Model 3 in Table 2. *P* for overall and P for nonlinearity are shown in the figure. A two-sided *P* < 0.05 was considered statistically significant. ALR, albumin-to-lymphocyte ratio; HR, hazard ratio; CI, confidence interval; RCS, restricted cubic spline.

Results of the RCS analysis are shown in Figure 3. A significant non-linear association was observed between ALR and the risk of 28-day mortality (*P* for nonlinearity < 0.001). At lower ALR levels, the hazard ratio declined rapidly with increasing ALR and reached its lowest point in the intermediate ALR range. Thereafter, the curve gradually increased. At higher ALR levels, the hazard ratio approached or slightly exceeded 1.

**Figure 4:**
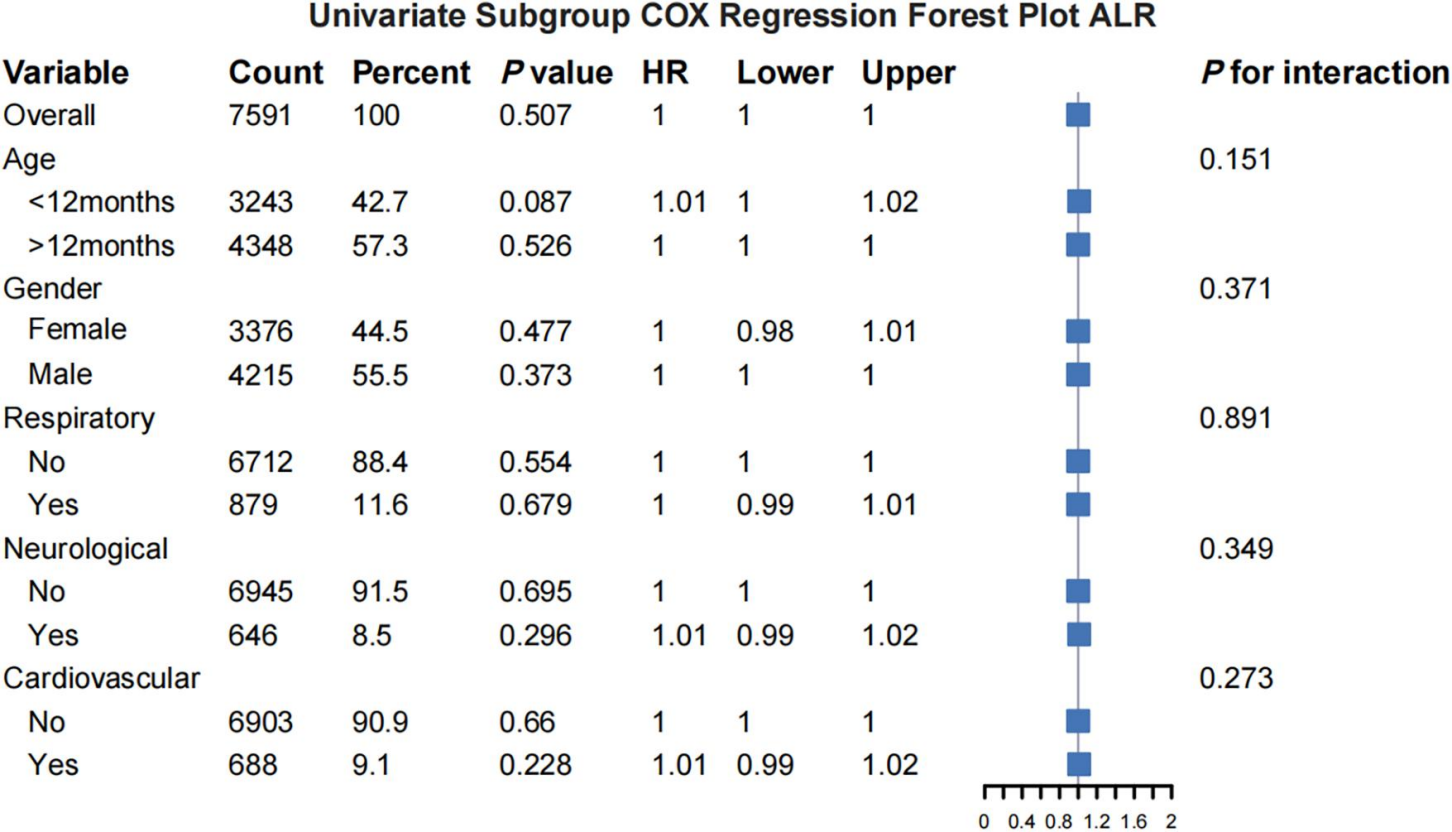
Association between ALR and 28-day mortality in pediatric patients across clinical subgroups using Cox regression. Forest plot showing subgroup analyses of the association between ALR and 28-day all-cause mortality. Squares represent hazard ratios (HRs), and horizontal lines represent 95% confidence intervals (CIs). P for interaction values are shown for each subgroup category. No significant interaction was observed if *P* for interaction was ≥ 0.05. A two-sided *P* < 0.05 was considered statistically significant. ALR, albumin-to-lymphocyte ratio; HR, hazard ratio; CI, confidence interval.

Subgroup analyses are presented in Figure 4. Overall, the association between ALR and outcome risk remained consistent across different clinical strata. The forest plot shows similar effect directions across subgroups, with substantial overlap of the 95 percent confidence intervals. No significant interactions were detected, suggesting the absence of marked heterogeneity. In addition, age, sex, and major diagnostic categories did not significantly modify this association. These findings suggest that ALR may serve as a stable indicator of short term prognosis in critically ill children and is relatively robust to differences in baseline clinical characteristics. In an exploratory ROC comparison of ALR alone with its individual components, ALR showed the highest AUC among ALR, albumin, and lymphocyte count [0.618 (95% CI 0.565–0.670) vs 0.562 (95% CI 0.508–0.616) and 0.480 (95% CI 0.429–0.531), respectively; Supplementary Figure S1].

### 3.3 Development and evaluation of the mortality risk prediction model

**Figure 5:**
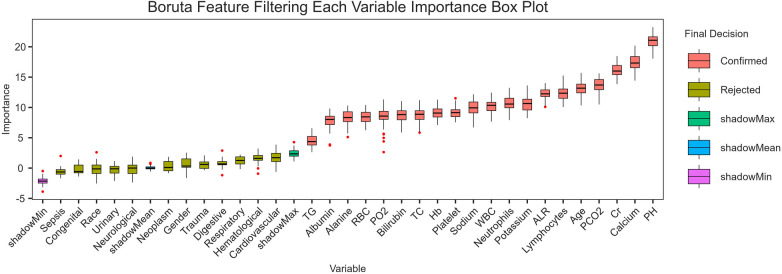
Variable selection for ALR-based mortality prediction in pediatric patients using the boruta algorithm. Boruta feature selection boxplot showing the relative importance of candidate predictors for 28-day all-cause mortality. Red boxplots indicate confirmed variables, yellow boxplots indicate rejected variables, and the shadow variables represent permuted reference features used by the Boruta algorithm (shadowMax, shadowMean, and shadowMin). Higher values indicate greater variable importance. A two-sided *P* < 0.05 was considered statistically significant for Boruta-confirmed features. ALR, albumin-to-lymphocyte ratio; TG, triglycerides; RBC, red blood cell count; PO2, partial pressure of oxygen; TC, total cholesterol; Hb, hemoglobin; WBC, white blood cell count; PCO2, partial pressure of carbon dioxide; Cr, creatinine; pH, potential of hydrogen.

 Figure 5 illustrates the results of feature selection using the Boruta algorithm. In the plot, red boxplots represent variables confirmed as important predictors, whereas yellow boxplots indicate variables that did not pass the selection process. Shadow features were included to provide a reference baseline. Ultimately, Boruta identified 20 predictors that were associated with 28 day all cause mortality.

**Figure 6:**
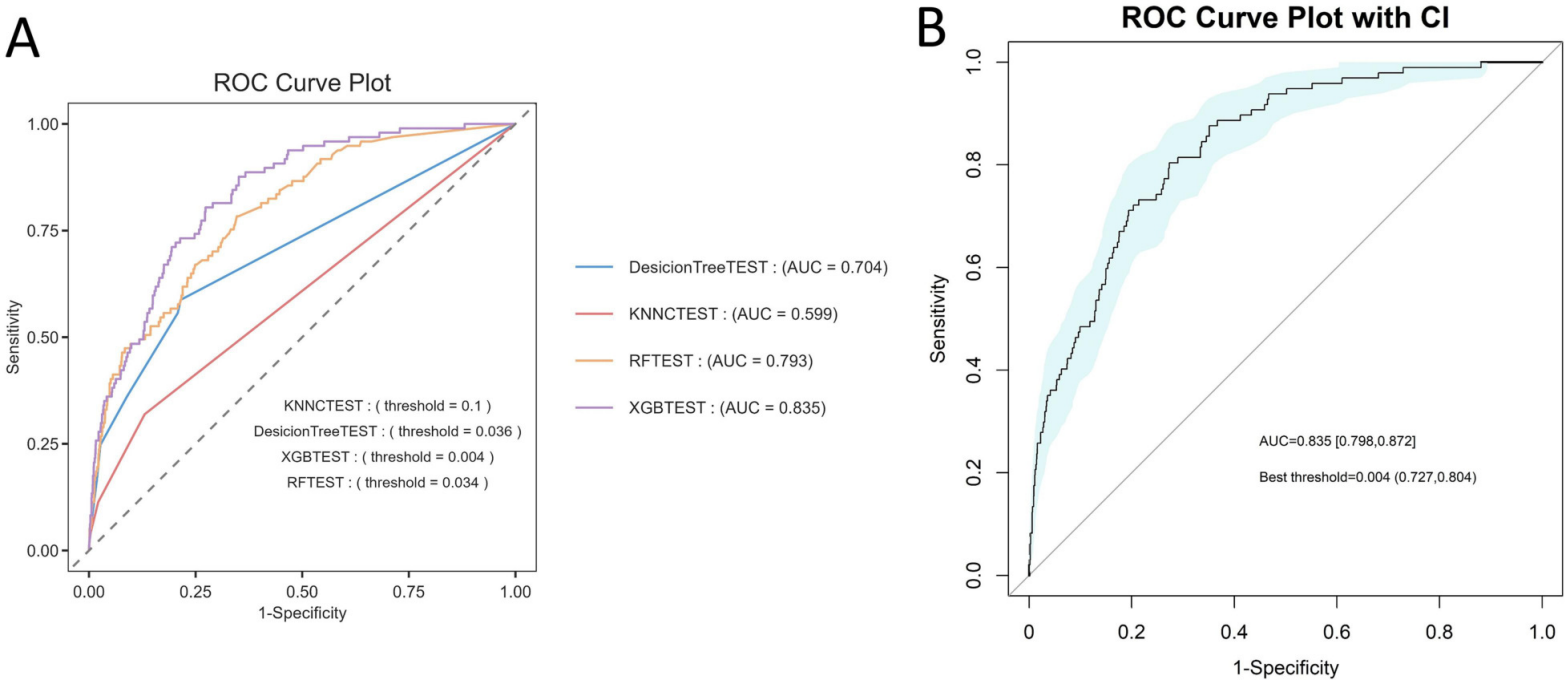
Prediction of 28-day mortality in pediatric patients using machine-learning discrimination analysis. **(A)** Receiver operating characteristic (ROC) curves of the evaluated machine-learning models in the testing set. **(B)** ROC curve with 95% confidence interval (CI) for the best-performing model, XGBoost, including the ROC-derived optimal cutoff and the corresponding sensitivity and specificity. Model performance was assessed using the area under the ROC curve (AUROC). The shaded area in panel B represents the 95% CI. A higher AUROC indicates better discrimination. DT, decision tree; KNNC, k-nearest neighbors classifier; RF, random forest; XGBoost, extreme gradient boosting; ROC, receiver operating characteristic; AUROC, area under the receiver operating characteristic curve; CI, confidence interval.

The predictive performance of the machine-learning models was evaluated in the testing set using multiple metrics. As shown in Figure 6A and Supplementary Table S2, XGBoost achieved the best overall performance among the evaluated models, with the highest AUROC (0.8351), the highest F1-score (0.2097), and the highest MCC (0.2381), followed by RF, decision tree, and KNNC. Because the SVM model yielded no positive predictions in the testing set and several threshold-dependent performance metrics were not estimable, it was not retained in the final comparative performance display. The ROC curve with 95% confidence interval for the XGBoost model is shown in Figure 6B. The AUROC was 0.835 (95% CI: 0.798–0.872). At the optimal cutoff identified from the ROC analysis, the sensitivity and specificity were 0.727 and 0.804, respectively. Decision curve analysis is shown in Supplementary Figure S2. Overall, the net benefit of the evaluated models was modest across most threshold probabilities. Precision–recall analysis further showed that XGBoost had the highest area under the PR curve among the compared models, suggesting relatively better identification of positive cases in this imbalanced dataset (Supplementary Figure S3). Calibration was additionally assessed using calibration plots and Brier scores (Supplementary Figure S4). Among the evaluated models, XGBoost showed the lowest Brier score (0.038), indicating relatively better overall calibration performance compared with the other models, although deviation from the ideal line remained in parts of the probability range.

**Figure 7:**
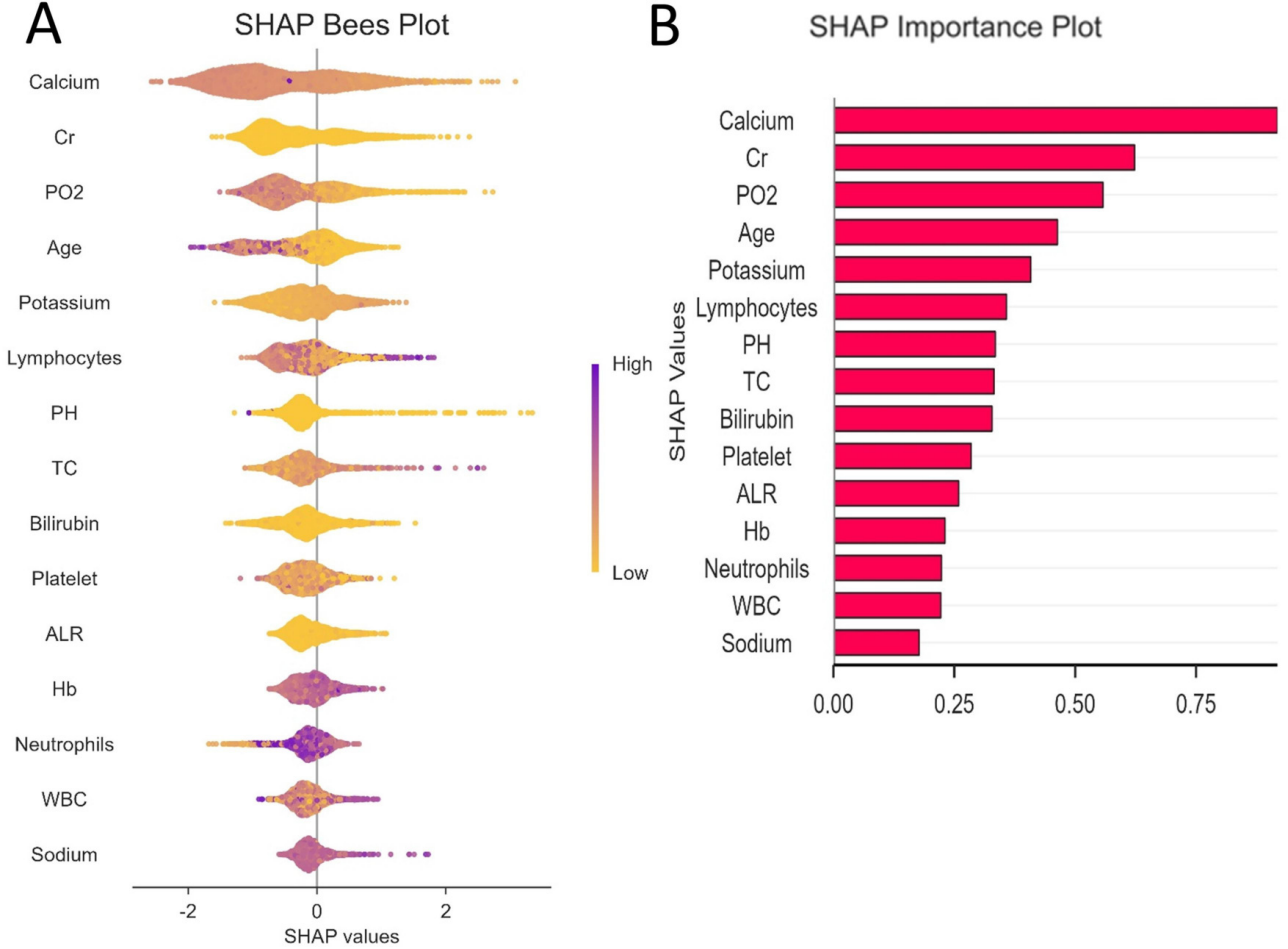
Contribution Of ALR to multivariable mortality prediction in pediatric patients using sHAP analysis. **(A)** SHAP beeswarm plot showing the distribution of SHAP values for each feature in the XGBoost model. Each dot represents one patient. Dot color reflects the relative feature value from low to high. The horizontal position indicates the SHAP value, that is, the direction and magnitude of that feature's contribution to the model prediction. Positive SHAP values indicate a greater contribution toward predicted mortality risk, whereas negative SHAP values indicate a lower contribution. **(B)** SHAP importance plot ranking variables according to the mean absolute SHAP values. Higher mean absolute SHAP values indicate greater overall importance in the model. ALR ranked tenth among the selected predictors. SHAP, Shapley additive explanations; ALR, albumin-to-lymphocyte ratio; Cr, creatinine; PO2, partial pressure of oxygen; pH, potential of hydrogen; TC, total cholesterol; Hb, hemoglobin; WBC, white blood cell count.

Figure 7 shows the SHAP-based interpretation of the predictive model. Figure 7A displays the SHAP beeswarm plot, which reflects both the direction and magnitude of each variable’s contribution at the individual level. Figure 7B presents the SHAP importance ranking according to the mean absolute SHAP values. ALR ranked tenth among the selected predictors, suggesting that it contributed to model predictions.

## 4 Discussion

This study systematically evaluated the association between the early ALR at admission and 28 day all cause mortality. The results showed clear differences in mortality across ALR tertiles. Survival curves indicated that patients with intermediate ALR levels had the highest survival probability, whereas those with low ALR had the poorest outcomes. In multivariable Cox regression analyses, the highest ALR tertile was consistently associated with a lower risk of 28 day mortality compared with the lowest tertile.RCS analysis further demonstrated a non linear relationship between ALR and mortality risk. At low ALR levels, the risk of death declined rapidly with increasing ALR, reached a nadir at intermediate values, and then gradually increased at higher levels. This pattern suggests that the prognostic meaning of ALR is not linear across its entire range: an increase from very low to intermediate ALR may reflect recovery from combined hypoalbuminemia and inflammatory catabolism, whereas further increases in ALR may increasingly be driven by reductions in lymphocyte count rather than by additional improvement in nutritional reserve. Subgroup analyses showed a consistent direction of effect across clinical strata, with no significant interactions observed, supporting the stability of the association between ALR and short term prognosis.In terms of risk prediction, the XGBoost model achieved the best discriminative performance in the testing set, with an AUC of 0.835. In addition, SHAP based interpretation ranked ALR among the most important features, indicating that ALR provides an independent contribution to individualized risk stratification.

After infection or tissue injury, critically ill children experience a rapid surge in inflammatory mediators, leading to a dynamic state in which hyperinflammation and immunosuppression may coexist (14). Lymphocyte apoptosis and functional exhaustion result in reduced peripheral lymphocyte counts, and persistent lymphopenia has been associated with multiple organ dysfunction and an increased risk of death (15). Pediatric sepsis is also characterized by marked heterogeneity, with distinct immune endotypes linked to different clinical outcomes (16). In hyperinflammatory syndromes such as multisystem inflammatory syndrome in children (MIS-C), immunomodulatory therapies targeting inflammatory pathways have been shown to shorten disease duration, underscoring the central role of inflammation driven injury (17). In addition, inflammation related disruption of the endothelial barrier can promote protein leakage and impair tissue perfusion (18). At the same time, critically ill children often rapidly enter a catabolic state dominated by undernutrition, with energy deficits and negative nitrogen balance developing early in the disease course. Even when body mass index appears normal at admission, nutritional indices may deteriorate during hospitalization and are associated with adverse outcomes, highlighting that dynamic nutritional decline should not be overlooked (19). In the setting of organ dysfunction, inadequate nutritional support is common, and insufficient protein and energy intake can impair muscle recovery and barrier repair, thereby prolonging convalescence (20). Large retrospective studies have shown that PICU patients with a diagnosis of malnutrition frequently have more underlying comorbidities, greater healthcare resource utilization, and a higher risk of readmission (21). Among children with complex chronic conditions, low body weight is associated with a greater burden of organ dysfunction and longer hospital stays, suggesting that limited physiological reserve amplifies the consequences of stress related catabolism (22). Serum albumin, as an integrated marker reflecting both nutritional status and inflammatory burden, has been shown to have a non linear association with PICU mortality. This finding supports the concept that hypoalbuminemia is not merely a marker of dilution or fluid overload, but may also reflect ongoing protein loss and microcirculatory impairment (23). Taken together, excessive immune inflammatory activation in critically ill children can continuously exacerbate tissue injury and drive the progression of organ dysfunction, while concomitant undernutrition further reduces protein reserves and weakens immune responses and repair capacity. These processes interact and reinforce each other, ultimately increasing the risk of short term adverse outcomes and mortality.

The ALR is composed of serum albumin and lymphocyte count and serves as a composite biomarker that reflects both nutritional reserves and immune inflammatory status. Our findings indicate that when ALR increases within the lower range, mortality risk declines rapidly. This pattern suggests that prognosis at low ALR levels is mainly driven by hypoalbuminemia related catabolism and increased vascular permeability. In pediatric critical illness, low albumin not only reflects inadequate energy and protein supply, but is also associated with inflammation mediated protein leakage, positive fluid balance, and greater requirements for organ support, all of which are closely linked to mortality (24). A systematic review and meta analysis further showed that malnutrition at PICU admission is associated with worse outcomes and a higher risk of death (25). In addition, the combination of lactate related metabolic stress and hypoalbuminemia in critical illness may further amplify the risk of adverse outcomes. Several albumin centered composite ratios have demonstrated consistent signals in predicting mortality in the pediatric ICU, which aligns with the sharp decline in risk observed at low ALR levels in our study (4, 26). As ALR enters the intermediate range, the risk curve tends to stabilize, possibly indicating partial buffering of nutritional deficits and inflammatory burden, with diminishing marginal benefit. Notably, the best prognosis in our cohort was observed in T2 rather than T3, suggesting that the prognostic effect of ALR may be optimal within an intermediate range instead of increasing monotonically across the entire spectrum. A plausible explanation is that patients in T2 may represent a relatively balanced immunonutritional state, in which albumin related reserve is less severely impaired while lymphocyte depletion has not yet become the dominant abnormality. Notably, the curve gradually rises at higher ALR levels, suggesting that reductions in the denominator, namely lymphocyte count, begin to dominate risk. Persistent lymphopenia or immune paralysis is common in critically ill children and is strongly associated with multiple organ dysfunction and increased mortality (27, 28). In infection related critical conditions such as sepsis, early alterations in immune cell profiles are closely linked to outcomes, and lymphocyte based indices can help identify high risk phenotypes. These observations provide clinical support for the rise in risk at higher ALR levels (29). Moreover, marked lymphopenia has also been reported in hyperinflammatory syndromes such as MIS-C and correlates with disease severity, further highlighting the broad relevance of lymphocyte imbalance to adverse pediatric outcomes (30). In our study, compared with T2, patients in T3 had only slightly higher albumin levels but markedly lower lymphocyte counts, indicating that very high ALR values may partly reflect severe lymphocyte depletion rather than a truly better biological condition. In this setting, the apparent increase in ALR may be driven more by immunosuppression-related lymphopenia than by further improvement in nutritional reserve, which may help explain why the protective effect was attenuated in T3. From a mechanistic perspective, inflammation and endothelial barrier injury can lead to protein loss, while lymphocyte apoptosis and exhaustion during the immunosuppressive phase impair host defense and tissue repair. The convergence of these two pathways may jointly shape the non linear risk pattern observed for ALR in this study (31, 32).

From a clinical application perspective, ALR may be more useful as an early bedside risk-stratification signal than as a stand-alone treatment trigger. In our cohort, the most favorable prognosis was observed in the intermediate tertile, whereas the highest mortality was observed in the lowest tertile. Therefore, a low ALR—particularly when it falls below the study-derived lower tertile threshold—may identify children who warrant more structured reassessment during the early stage of PICU admission. In such patients, clinicians may consider formal nutritional risk evaluation, assessment of the feasibility of early enteral nutrition when not contraindicated, review of fluid balance and capillary leak, repeat measurement of albumin and lymphocyte count, and closer surveillance for sepsis, organ dysfunction, and immune dysregulation. Conversely, very high ALR values should not be interpreted as uniformly reassuring. In some patients, a markedly elevated ALR may be driven predominantly by lymphocyte depletion rather than by improved physiological reserve. In this setting, review of the absolute lymphocyte count, infection burden, and immune status, with intensified monitoring when clinically indicated, may be reasonable. To enhance the clinical interpretability of our findings, a proposed bedside workflow for ALR-based early risk assessment is provided in Supplementary Figure S5. Importantly, this workflow is intended to support risk stratification rather than replace clinical judgment, and it should not be interpreted as a validated ALR-guided treatment algorithm.

In summary, ALR captures both nutritional reserves and immune effector status within a single metric in critically ill children. In this study, ALR showed a non linear association with short term mortality. Low ALR reflected the coexistence of impaired immune defense and depleted protein reserves, which may predispose patients to infectious complications, worsening organ dysfunction, and reduced repair capacity under intense inflammatory stress, ultimately leading to poorer outcomes. As ALR increased, mortality risk declined rapidly within the lower range and reached its lowest point at intermediate levels, suggesting that improvements in immunonutritional status confer substantial benefit. However, with further increases in ALR, the protective association was attenuated, and the curve flattened or even showed a slight rebound, indicating that the best prognosis was observed at intermediate rather than highest ALR levels. This pattern may reflect that, in some patients, very high ALR values are driven predominantly by lymphocyte depletion rather than by meaningful improvement in albumin related reserve, so that a higher ratio does not necessarily correspond to better physiological status. Therefore, an intermediate ALR level may reflect a more balanced clinical state, whereas excessively high ALR should be interpreted with caution because it may indicate clinically relevant lymphopenia and impaired immune competence. Overall, ALR is derived from routine laboratory tests, is easy to calculate, and can be repeatedly measured, making it well suited for early risk stratification and identification of high risk patients at PICU admission. It may also serve as a complementary marker for dynamic monitoring of physiological status.

Several limitations of this study should be acknowledged. First, this was a retrospective single center cohort study. Despite multivariable adjustment and the use of machine learning models, residual confounding and selection bias cannot be excluded, and causal inferences should therefore be made with caution. Second, some key variables were missing or not available in the database, such as detailed cytokine profiles, immune phenotyping, and comprehensive nutritional assessment tools. This limited our ability to more precisely elucidate the immunometabolic mechanisms underlying changes in ALR. Third, the study population was drawn from a specific region and healthcare system. The generalizability of the findings to other countries, ethnic groups, or PICU settings with different resource levels requires further validation in multicenter prospective studies. Finally, although we supplemented model evaluation with PR analysis, calibration plots, Brier scores, a comparative performance table, and ROC-derived sensitivity and specificity for the best-performing model, some additional calibration and threshold-based measures, such as the Hosmer–Lemeshow test and predictive values at the ROC-derived optimal threshold, were not robustly obtainable in the current analytical workflow. Therefore, the clinical applicability of the model should be interpreted with caution and requires further validation.In addition, the threshold-based clinical interpretation and the proposed workflow derived from the present study are exploratory and require prospective multicenter validation before being used to guide bedside management.

## Conclusion

ALR was independently associated with short term all cause mortality in critically ill pediatric patients. Models that integrated ALR with other clinical features showed good discrimination for mortality prediction, supporting the added value of ALR in risk assessment. As a simple and low cost biomarker derived from routine laboratory tests, ALR may aid early risk stratification and prognostic evaluation in the PICU.

## Data Availability

The original contributions presented in the study are included in the article/Supplementary Material, further inquiries can be directed to the corresponding author.
