# Supplementary material for: Association between the albumin-to-lymphocyte ratio and short-term mortality in critically Ill pediatric patients: a retrospective cohort study and machine learning analysis
Source: Front Pediatr. 2026 May 25;14:1795057. doi: 10.3389/fped.2026.1795057 (PMC13243273; doi:10.3389/fped.2026.1795057)
Supplement: Supplementary file 1 [file Supplementaryfile1.docx]

**Supplementary materials**

**Supplementary Table 1** All Variables extracted from the PIC Database.

| **Category** | **Variables Included** |
| --- | --- |
| Demographics | Age, Gender, and Race. |
| Laboratory indicators | Hemoglobin, WBC, Lymphocyte count, Platelet, RBC, Neutrophils, Potassium, Sodium, Calcium, Albumin, PCO2, PH, PO2, TG, TC, Alanine, Cr, and Bilirubin. |
| Underlying comorbid conditions | Sepsis, Urinary diseases, Trauma, Respiratory diseases, Neurological diseases, Neoplasms, Hematological diseases, Digestive diseases, Congenital diseases, Cardiovascular diseases. |
| Clinical outcomes | 28-day hospital Mortality. |
| ALR, Albumin-to-Lymphocyte Ratio; WBC, White Blood Cell count; RBC, Red Blood Cell count; TG, Triglycerides; TC, Total Cholesterol; Cr, Creatinine; PCO2, Partial Pressure of Carbon Dioxide; PO2, Partial Pressure of Oxygen; PH, pH (Potential of Hydrogen); ICU, Intensive Care Unit; PICU, Pediatric Intensive Care Unit; CICU, Cardiac Intensive Care Unit; NICU, Neonatal Intensive Care Unit; SICU, Surgical Intensive Care Unit. | |

**Supplementary Table 2** Overall performance comparison of machine-learning models in the testing set.

| **Model** | **AUROC** | **Sensitivity** | **Specificity** | **Accuracy** | **F1-score** | **MCC** |
| --- | --- | --- | --- | --- | --- | --- |
| XGBoost | 0.8351 | 0.134 | 0.9936 | 0.957 | 0.2097 | 0.2381 |
| Random forest | 0.7933 | 0.0309 | 0.9991 | 0.9579 | 0.0588 | 0.1295 |
| Decision tree | 0.7041 | 0.0206 | 0.9991 | 0.9574 | 0.0396 | 0.095 |
| KNNC | 0.5987 | 0.0412 | 0.9963 | 0.9557 | 0.0734 | 0.1048 |
| AUROC, area under the receiver operating characteristic curve; MCC, Matthews correlation coefficient; KNNC, k-nearest neighbors classifier; XGBoost, extreme gradient boosting. | | | | | | |

**Supplementary Figure 1** ROC comparison of ALR alone, albumin alone, and lymphocyte count alone for predicting 28-day mortality in pediatric patients.


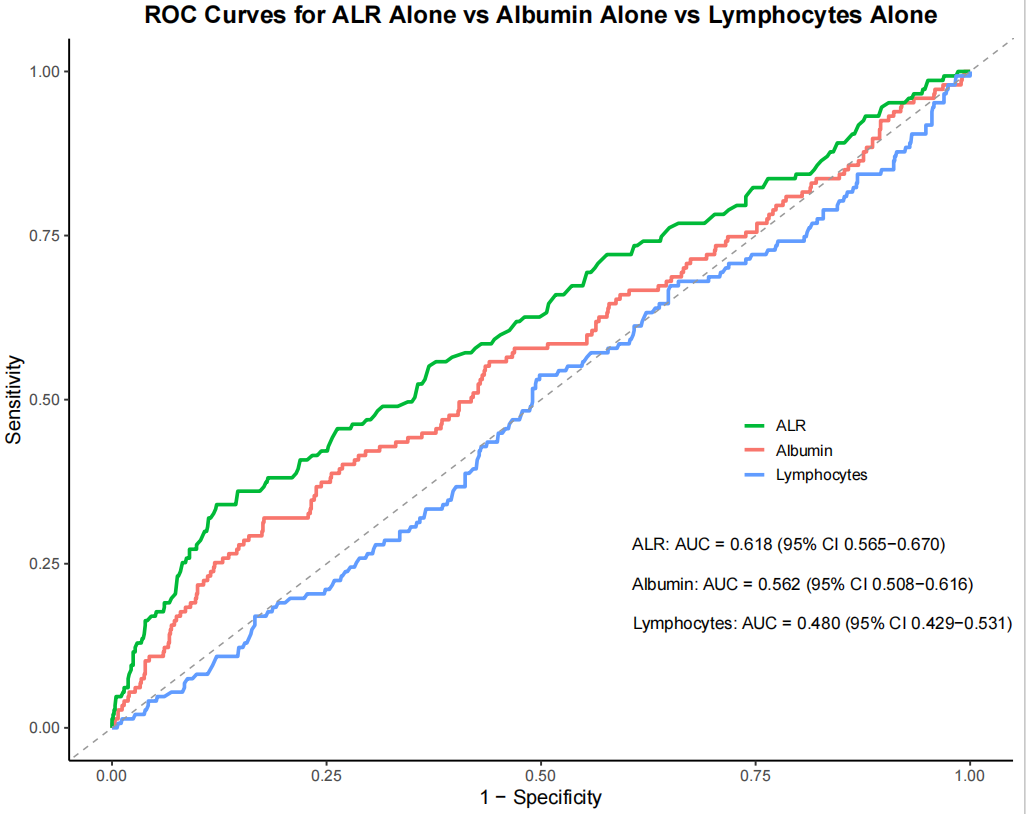


Footnote: Receiver operating characteristic (ROC) curves comparing the discrimination of ALR alone, albumin alone, and lymphocyte count alone for 28-day all-cause mortality. The area under the ROC curve (AUC) and 95% confidence interval (CI) are shown for each marker. This analysis is exploratory and intended to compare the composite index with its individual components. AUC, area under the receiver operating characteristic curve; CI, confidence interval; ALR, albumin-to-lymphocyte ratio.

**Supplementary Figure 2** Net clinical benefit of ALR-based machine-learning models using decision curve analysis.


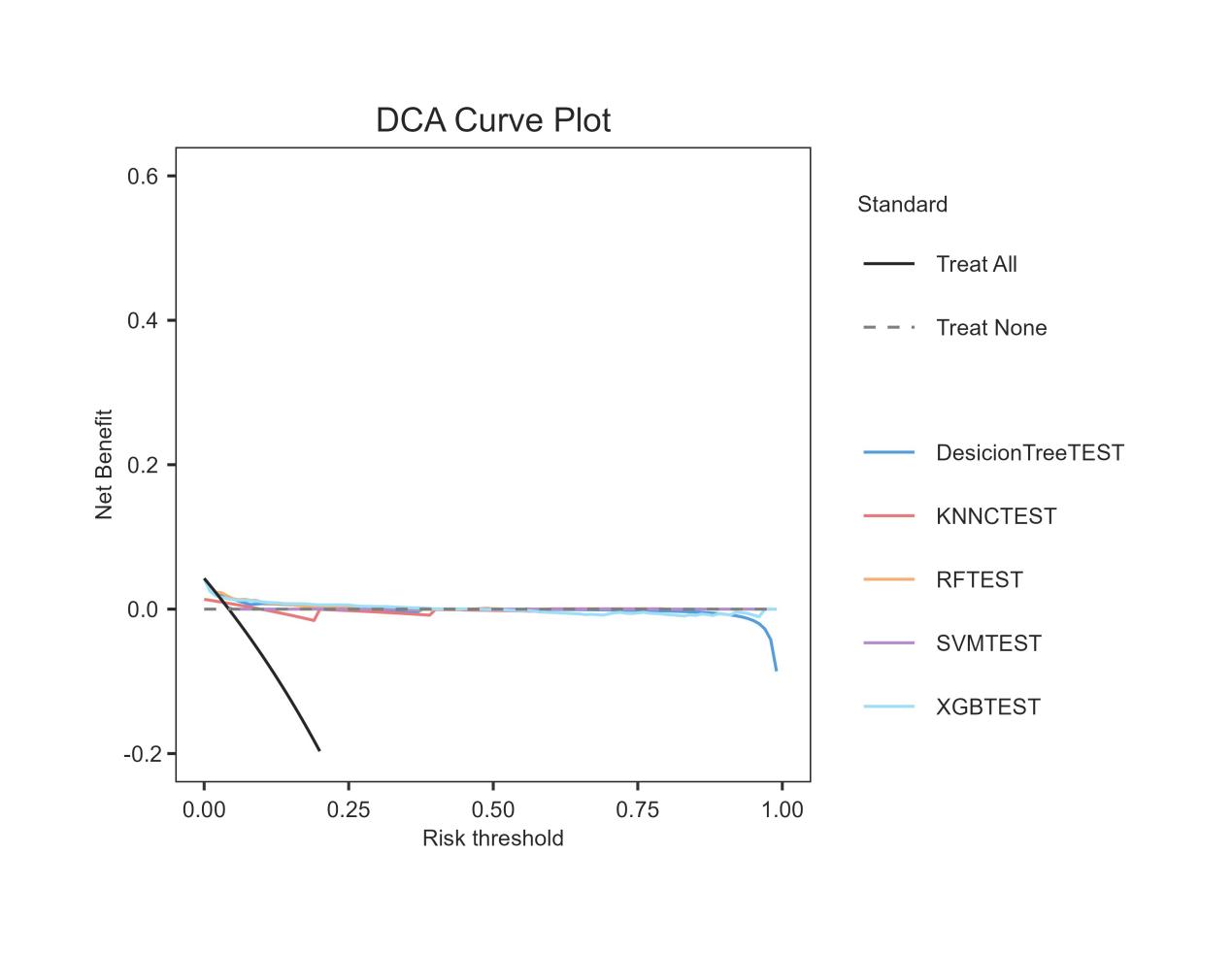


Footnote: Decision curve analysis (DCA) showing the net benefit of the evaluated machine-learning models across a range of threshold probabilities. The solid black line represents the “treat all” strategy, and the dashed black line represents the “treat none” strategy. Higher net benefit indicates greater potential clinical utility. DCA, decision curve analysis; DT, decision tree; KNNC, k-nearest neighbors classifier; RF, random forest; XGBoost, extreme gradient boosting.

**Supplementary Figure 3** Precision–recall performance of ALR-based machine-learning models for predicting 28-day mortality in pediatric patients.


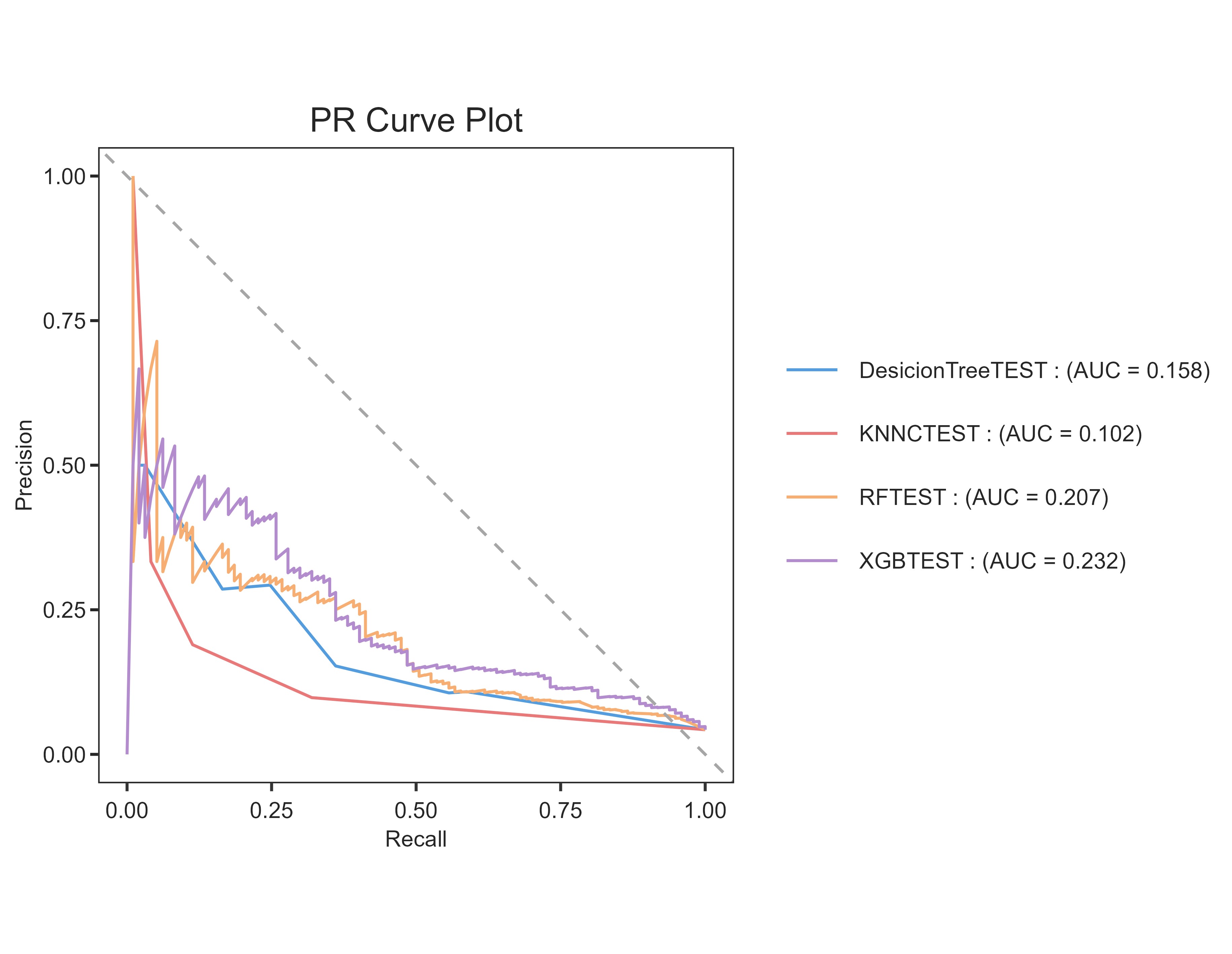


Footnote: Precision–recall curves of the evaluated machine-learning models in the testing set. This analysis complements ROC analysis in the setting of imbalanced outcome data. The AUC values shown in the figure correspond to the area under the precision–recall curve. PR, precision–recall; AUC, area under the curve; DT, decision tree; KNNC, k-nearest neighbors classifier; RF, random forest; XGBoost, extreme gradient boosting.

**Supplementary Figure 4** Calibration performance of ALR-based machine-learning models for predicting 28-day mortality in pediatric patients.


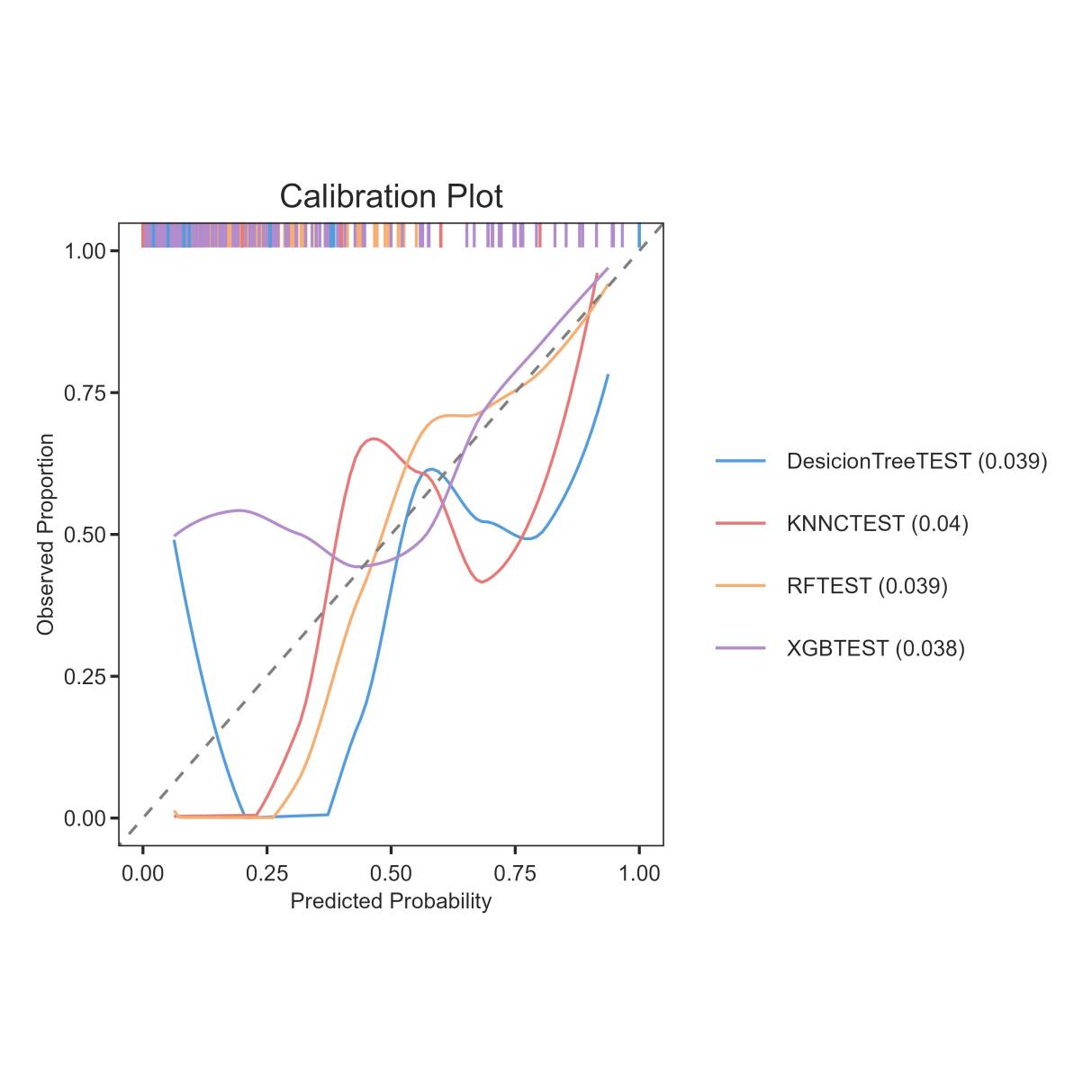


Footnote: Calibration plots comparing predicted and observed risk in the testing set. The diagonal dashed line represents ideal calibration. Values in parentheses indicate Brier scores, with lower values indicating better overall calibration performance. Calibration was assessed for the evaluated machine-learning models, and XGBoost showed the lowest Brier score. DT, decision tree; KNNC, k-nearest neighbors classifier; RF, random forest; XGBoost, extreme gradient boosting

**Supplementary Figure 5** Proposed clinical workflow for integrating ALR into early bedside risk assessment in critically ill pediatric patients.


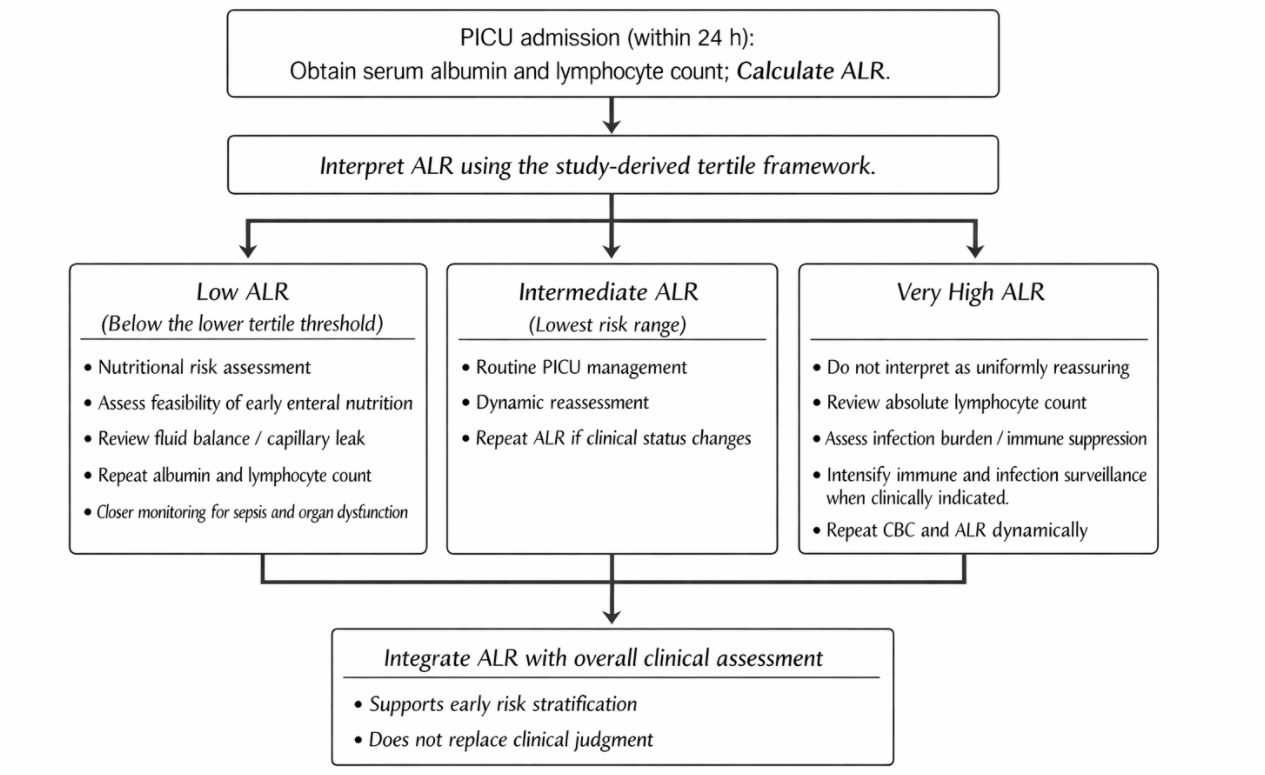


Footnote: Proposed bedside workflow illustrating how ALR may be incorporated into early risk stratification after PICU admission. Low ALR may prompt enhanced nutritional evaluation and closer monitoring, whereas very high ALR may prompt assessment for lymphopenia-dominant immune dysfunction. This workflow is exploratory and hypothesis-generating and should not be interpreted as a validated ALR-guided treatment algorithm. CBC, complete blood count; ALR, albumin-to-lymphocyte ratio; PICU, pediatric intensive care unit.
